# Survey data on entrepreneurs׳ subjective plan and perceptions of the likelihood of success

**DOI:** 10.1016/j.dib.2016.01.034

**Published:** 2016-01-30

**Authors:** Quan Hoang Vuong

**Affiliations:** Centre Emile Bernheim, Solvay Brussels School of Economics and Management, Université Libre de Bruxelles, 50 Ave. F.D. Roosevelt, Brussels B-1050, Belgium

**Keywords:** Entrepreneurship, Vietnam, Likelihood of success, Entrepreneurial attempt, Socioeconomic conditions, Transition economy

## Abstract

Entrepreneurship is an important economic process in both developed and developing worlds. Nonetheless, many of its concepts appear to be difficult to ‘operationalize’ due to lack of empirical data; and this is particularly true with emerging economy. The data set described in this paper is available in Mendeley Data׳s “Vietnamese entrepreneurs’ decisiveness and perceptions of the likelihood of success/continuity, Vuong (2015) [1]” http://dx.doi.org/10.17632/kbrtrf6hh4.2; and can enable the modeling after useful discrete data models such as BCL.

**Specifications Table**

**Table:** 

Subject area	*Economics*
More specific subject area	*Business Economics/Entrepreneurship*
Type of data	*Table, text file, graph*
How data was acquired	*Survey*
Data format	*Raw, filtered, and partially analyzed*
Experimental factors	*Raw data obtained from direct survey on participants of seminars, conferences and meetings. Incomplete data sheets are eliminated.*
Experimental features	*The experiment focuses on perceptions and subjective understanding of prospective and extant entrepreneurs in Vietnam.*
Data source location	*Hanoi, Ho Chi Minh City, Buon Ma Thuot, Da Nang of Vietnam*
Data accessibility	*Datasets are provided with this article.* Mendeley Data, v2, http://dx.doi.org/10.17632/kbrtrf6hh4.2

**Value of the data**

• The data offer an opportunity to measure the decisiveness and preparedness of an entrepreneur given various conditions that characterize an emerging market.
• Information and deeper insights that might be obtained through discrete data analysis can help predict behaviors of entrepreneurs in typical situations, and formulate policy responses if the government wishes to improve the business/economic environment.
• Important aspects of entrepreneurship such as creativity/innovation, previous professional experience, personal perceptions of socio-cultural values, and the like can be researched and later ‘operationalized’.
• The data reflect the transition of the emerging market economy of Vietnam.

## 1 Data

The data set contains 3071 records obtained from a nationwide survey of perceptions, intentions and assessments from entrepreneurs, existing and prospective, about the socio-economic conditions, values of their previous employment, need of government- and society-supported entrepreneurship-enabling programs. The data also provide subjective evaluation of the likelihood of success or continuity of entrepreneurs׳ project given certain environmental conditions. The following discrete (categorical) variables are measured in the survey:

**Table:** 

Coded name	Explanation	Values
X1.job	Previous job (or the most important)	Human resources (hmr); sales/marketing (salesm), production/operations (pom); finance/accounting (finance); administrative or other departments (admin); no significant job experience (none)
X2.starthis	Entrepreneurial status	Running, dropped, notyet
X3.econdf	Assessment on whether current socio-economic conditions are favorable.	Favorable (fav); somewhat favorable (somewhat); and, unfavorable (unfav)
X4.suppval1	Influence of government or society-funded supports on decision?	High; avg; low
X5.suppval2	Influence of government or society-funded entrepreneurship programs on success?	High; possible; none
X6.infpeople	Influence from family, friends, colleagues.	Strong; somewhat; light; none
X7.tforstart	How much time for this entrepreneurial attempt?	Less than 12 months (less12); 12–24 months (b1224); and, until success (g24)
X8.tforrest	Extra time an entrepreneur gives himself/herself when the project goes bad?	Less than 12 months (less12); 12–24 months (b1224); and, until success (g24)
X9.member	Participation in professional societies, entrepreneurs forums, online networks?	As many as possible (all); in case forums/associations but selective (some); and, none.
X10.failurel	Learning from others׳ failures?	Careful study (a); exploring few noteworthy cases (b); and, no need (c).
X11.plan	Having a business plan?	A good one (good); a basic plan (basic); in process of making one (inprocess); and, having no need right now (noneed)
X12.resconst	Most serious constraint?	Finance; product; relationship; management skills; technical; others.
X13.mres	Resources for startup	Relatives/friends; investors; gov (donors and government); saving; and, none.
X14.inno	Self-evaluation of creativeness of product/services/business model?	Creative (a), somewhat creative (b), hopefully (c); not at all (d)
X15.diff	Self-evaluation of differentiation of product/services/business model?	Strong (a); Significant differentiation (b); Somewhat different (c); and, not at all (d)
X16.tot1strev	Time to the first dollar (revenue)?	Currently generating revenues (a); in 6 months (b); 6–12 months (c); uncertain (d).
X17.keystrength	Most decisive strength for success	Sales skills (a); good products/services (b); efficient business models (c); accurate prediction of market trend (d); hard working (e); and, patience/tenacity (f).
X18.startplan	When deciding to start own business?	Operating (a); soon (b); within 12 months (c); when conditions are favorable (d); perhaps never (e).
X19.msponge	Entrepreneurial efforts transform ways of thinking, acting and beliefs?	Strong; some aspects; negligible; none
X20.bplantime	When a strategy is needed?	When project begins (a); having revenue (b); firm growing (c); unnecessary (d)
X21.restart	If this turns out a failure, another entrepreneurial attempt?	Never; when possessing new resources; when having new/better ideas; when market conditions and policies support entrepreneurial plan.
X22.csr	Helping next generations of entrepreneurs?	No (a); Yes if that helps improve sales (b); Yes, unconditionally (c).
X23.chance	Evaluated chance of success?	Certain; high (>80%); med (50–80%); low (<50%).
Sex	Gender	Male/Female

## 2 Experimental design, materials and methods

The survey was designed to obtain discrete data that can be employed by the multi-category logit models to enable analysis based on baseline-category logits (BCL), which helps provide estimated coefficients for computing probabilities upon events of hypothetical influence. The logic for designing the experiment and thus data set is described as follows. For designing both the survey and prepare the data set and suitable subset, an entrepreneur (among *n*) is treated as independent and identical. Each data point has outcome in any of *J* categories for each factor to be investigated. Let *y*_*ij*_=1 if entrepreneur *i* has outcome in category *j* and *y*_*ij*_=0 therwise. Then, $y_{ij}=\left(y_{i1},y_{i2},\ldots ,y_{ic}\right)$ represents a multinomial trial, with $\sum_{j}y_{ij}=1$. As $n_{j}=\sum_{j}y_{ij}$ the number of trials having outcome in category *j*, the design is based on the assumption that $\left(n_{1},n_{2},\ldots ,n_{c}\right)$ show a multinomial distribution. Let *π*_*j*_=*P*(*Y*_*ij*_=1) denote the probability of outcome in category *j* for each entrepreneur, the multinomial PMF is:

$$p\left(n_{1},n_{2},\ldots ,n_{c}\right)=\left(\frac{n!}{n_{1}!n_{2}!\cdots n_{c}!}\right)\pi_{1}^{n_{1}}\pi_{2}^{n_{2}}\cdots \pi_{c}^{n_{c}},$$

with:

$$E\left(n_{j}\right)=n\pi_{j}\;\mathrm{var}\left(n_{j}\right)=n\pi_{j}\left(1-\pi_{j}\right)\;\mathrm{cov}\left(n_{j},n_{k}\right)=-n\pi_{j}\pi_{k}$$

where $\sum_{j}n_{j}=n$. As $\pi_{j}\left(x\right)=P\left(Y=j|x\right)$ and $\sum_{j}\pi_{j}\left(x\right)=1$, data are grouped into *J* categories of *Y* as multinomial with corresponding sets of probabilities $\left\{\pi_{1}\left(x\right),\ldots ,\pi_{j}\left(x\right)\right\}$. Thus, each response is aligned with a baseline category:

$$\ln\;\frac{\pi_{j}\left(x\right)}{\pi_{J}\left(x\right)}=\alpha_{j}+\beta_{j}^{\prime }x,\;\;j=1,\ldots ,J-1.$$

BCL analysis simultaneously models the effects of **x** on (*J*−1) logits, which in general vary according to the response paired with the baseline category. The estimating of (*J*−1) equations employing a given empirical data set would provide for parameters for these logits, as:

$$\ln\;\frac{\pi_{a}\left(x\right)}{\pi_{b}\left(x\right)}=\;\ln\;\frac{\pi_{a}\left(x\right)}{\pi_{J}\left(x\right)}-\;\ln\;\frac{\pi_{b}\left(x\right)}{\pi_{J}\left(x\right)}.$$

The empirical data set enables the computing of Pearson-type likelihood ratio test statistics (*X*^2^, *G*^2^) for goodness-of-fit, following a multivariate generalized linear model (GLM) estimations:

$$g\left(\mu_{i}\right)=X_{i}\beta ,$$

where, $\mu_{i}=E\left(Y_{i}\right)$, corresponding to $y_{i}=(y_{i1},y_{i2},\ldots )\prime$; row *h* of the model matrix **X**_*i*_ for observation *i* contains values of independent variables for *y*_*ih*_. For a BCL model, $y_{i}=(y_{i1},y_{i2},\ldots ,y_{i,J-1})\prime$; *y*_*iJ*_ is redundant, thus:

$$\mu_{i}=\left(\pi_{1}\left(x_{i}\right),\pi_{2}\left(x_{i}\right),\ldots ,\pi_{J-1}\left(x_{i}\right)\right)\;g_{j}\left(\mu_{i}\right)=\;\ln\left\{\mu_{ij}/\left[1-\left(\mu_{1}+\cdots +\mu_{i,J-1}\right)\right]\right\}.$$

Technical details for practical modeling of polytomous logistic models is provided in [2]. Applied analysis can be performed in R (see [3]). Practical uses of survey data can be referred to [4].

**Fig. 1:**
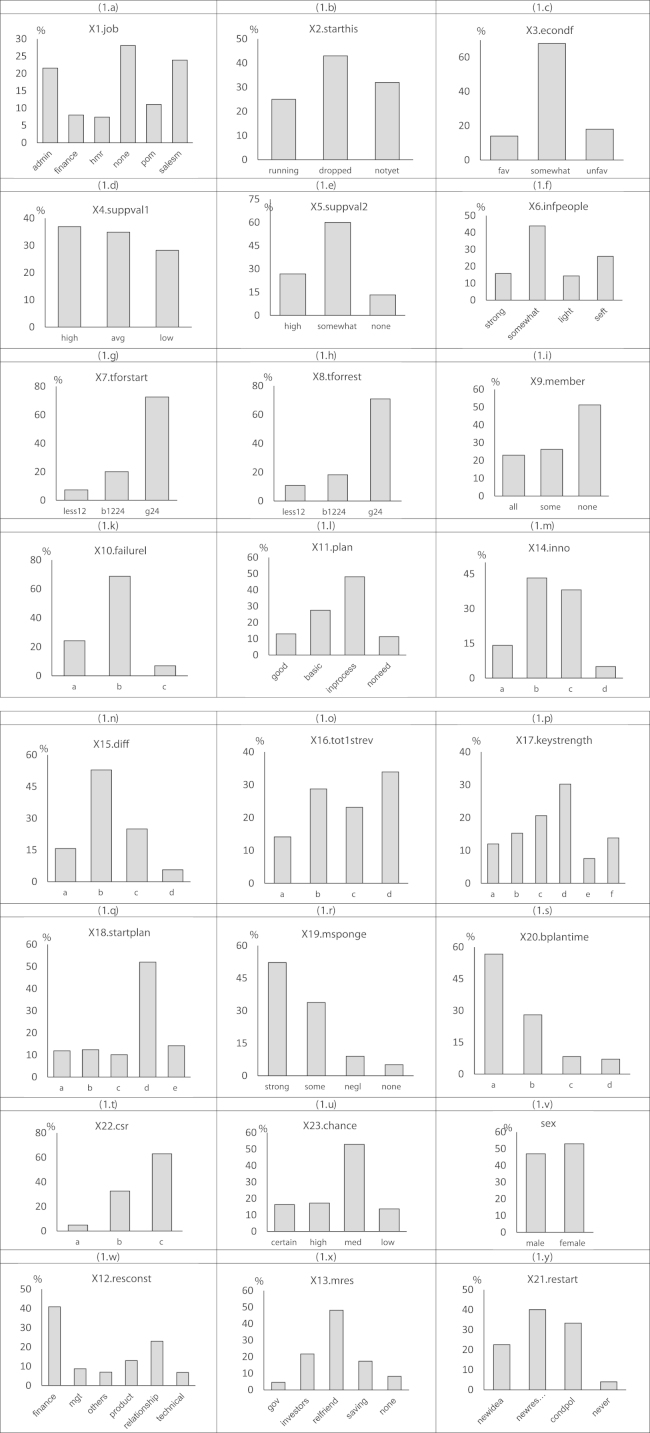
Some graphs from the raw data.

Explanation of data subsets filtered for different analysis purposes (from [1]) (Fig. 1).

**Table:** 

File name	Filtered with variables:	Frequency distributions table	Appropriate for hypothesis testing of:
x14.15.23	• “X14.inno” • “X15.diff” • “X23.chance”	gt1.1	Creativity and differentiation (entrepreneurs׳ products/services) influence entrepreneurs׳ perceptions of the likelihood of success/continuity.
x3.5.23	• “X3.econdf” • “X5.suppval2” • “X23.chance”	gt1.2	Entrepreneurs׳ assessments on economic conditions and environmental factors (such as support programs) and their impacts on the likelihood of success/continuity.
xsex.7.18	• “sex” • “X7.tforstart” • “X18.startplan”	gt2.1	Gender and strategic intent of timing and duration of efforts by entrepreneurs, in conjunction with their final decision.
x1.3.15.18	• “X1.job” • “X3.econdf” • “X15.diff” • “X18.startplan”	gt2.2	The impact of entrepreneurs׳ past employment together with self-assessmet of economic conditions, product innovations on the startup decision and likely continuity.
x1.9.15.18	• “X1.job” • “X3.econdf” • “X9.member” • “X18.startplan”	gt2.3	The impact of entrepreneurs׳ past employment together with self-assessmet of economic conditions, and their networks on the ultimate decisions of starting up and likely continuity.
x11.23.7	• “X11.plan” • “X23.chance” • “X7.tforstart”	gt2.4	Impacts of entrepreneurial planning and perceptions on chance of survival on the timing and likelihood of entrepreneurial undertaking/continuity.

One example of the analysis is to compute response probabilities from multinomial logits, i.e. $\left\{\pi_{j}\left(x\right)\right\}$, using $\pi_{j}\left(x\right)=\frac{\exp\left(\alpha_{j}+\beta_{j}^{\prime }x\right)}{1+\sum_{h=1}^{J-1}\exp\left(\alpha_{h}+\beta_{h}^{\prime }x\right)}$; with $\sum_{j}\pi_{j}\left(x\right)=1$; *α*_*J*_=0 and **β**_*J*_=0. An empirical distribution is provided in Table 1.

**Fig. 2:**
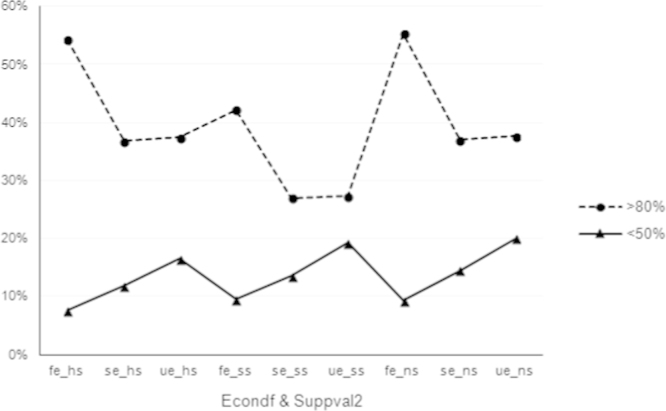
Evaluated chance of success based on economic conditions and values of support.

Picking two different trends, the contrast shown by the empirical data becomes apparent in Fig. 2, suggesting that, if a government aims to promote entrepreneurship, it is better to improve general socio-economic conditions.

## Figures and Tables

**Table 1:** Conditional probabilities of entrepreneurial success based on socio-economic conditions and perceived values of government-funded or society-promoted supporting programs.

X23.Chance	Certain			High			Med			Low		
X3.econdf| X5.suppval2	High	s/w	None	High	s/w	None	High	s/w	None	High	s/w	None
Fav	0.335	0.232	0.355	0.208	0.19	0.198	0.381	0.482	0.354	0.076	0.096	0.093
Somewhat	0.17	0.106	0.18	0.198	0.164	0.19	0.514	0.594	0.485	0.118	0.136	0.145
Unfav	0.207	0.132	0.218	0.168	0.141	0.158	0.46	0.535	0.424	0.165	0.192	0.2
