# Supplementary material for: Survey data on entrepreneurs׳ subjective plan and perceptions of the likelihood of success
Source: Data Brief. 2016 Jan 30;6:858–64. doi: 10.1016/j.dib.2016.01.034 (PMC4749942; doi:10.1016/j.dib.2016.01.034)
Supplement: Supplementary file 1 — Supplementary data [file mmc1.pdf]

AUTHOR DECLARATION TEMPLATE

I wish to confirm that there are no known conflicts of interest associated with this publication and there has been no significant financial support for this work that could have influenced its outcome.

I confirm that the manuscript has been prepared by myself.

I confirm that I have given due consideration to the protection of intellectual property associated with this work and that there are no impediments to publication, including the timing of publication, with respect to intellectual property. In so doing I confirm that I have followed the regulations and laws of my institutions and my country concerning intellectual property.

I understand that the Corresponding Author is the sole contact for the Editorial process (including Editorial Manager and direct communications with the office). I confirm that I have provided a current, correct email address which is accessible by the Corresponding Author and which has been configured to accept email from qvuong@ulb.ac.be

Signed by Quan Hoang Vuong, Ph.D.

Vuong Quan Hoang

Dec 3 2015
